# Dynamic transcriptome analysis of NFAT family in guided bone regeneration with occlusive periosteum in swine model

**DOI:** 10.1186/s13018-022-03252-9

**Published:** 2022-07-26

**Authors:** Bao-Fu Yu, Ning Yin, Zi Wang, Xiao-Xue Chen, Chuan-Chang Dai, Jiao Wei

**Affiliations:** 1 Department of Plastic and Reconstructive Surgery, Shanghai Ninth People’s Hospital Affiliated to Shanghai Jiaotong University School of Medicine, No. 639 Zhi Zao Ju Road, Shanghai, 200011 China; 2 Department of Ear-Nose-Throat, Eye and ENT Hospital of Fudan University, Shanghai, China

**Keywords:** Bone defects, Guided bone regeneration, Periosteum, NFAT family, Gene dynamic expression

## Abstract

**Objective:**

To investigate the dynamic expression of NFAT family of periosteum in guided bone regeneration process.

**Material and methods:**

The swine ribs on one side were used as the trauma group and the contralateral side as the control group. After rib segment was removed, periosteum was sutured to form a closed cavity mimicking guided bone regeneration. The periosteum and regenerated bone tissue were collected at nine time points for gene sequencing and hematoxylin–eosin staining. The expression data of each member were extracted for analysis. Expression correlations among various members were analyzed.

**Results:**

Staining showed the guided bone regeneration was almost completed 1 month after the operation with later stage for bone remodeling. The expression levels of each member in both groups changed greatly, especially within postoperative 1.5 months. The expression of NFATc1 and NFATC2IP in trauma group was significantly correlated with those of control group. The foldchange of each member also had large fluctuations especially within 1.5 months. In the trauma group, NFATc2 and NFATc4 were significantly upregulated, and there was a significant aggregation correlation of NFAT family expression between the various time points within one month, similar to the “pattern-block” phenomenon.

**Conclusion:**

This study revealed the dynamic expression of NFAT family in guided bone regeneration, and provided a reference for the specific mechanism. The first 1.5 months is a critical period and should be paid attention to. The significant high-expression of NFATc2 and NFATc4 may role importantly in this process, which needs further research to verify it.

**Supplementary Information:**

The online version contains supplementary material available at 10.1186/s13018-022-03252-9.

## Introduction

Large bone and joint defects are not rare, and they are clinically difficult for treatment. Based on the principle of guided tissue regeneration which takes advantage of the cell with capacity to regenerate the certain type of tissue [1], the guided bone regeneration was proposed as a new treatment for bone and joint defects, which induces bone formation-related cells for metabolism and perform bone tissue regeneration by forming a closed bone regeneration microenvironment [2–4]. The key factor in this procedure is the closed sheath created by osteogenic cell populations [3]. In previous clinical studies, bone tissue regeneration can usually be combined with metal implants and bone grafts to repair large bone defects [5]. Critical size osseous defects in maxillofacial surgery including maxillofacial and calvarial defects have been successfully reconstructed [3, 6]. Although there are many studies on the related molecular mechanisms of fracture healing, there are few studies on the related molecular mechanisms of guided bone regeneration and most of them are in the preliminary exploration stage.

In our previous studies, we have used bone tissue regeneration technique to reconstructed large mandibular defects and temporomandibular joint defects in goat model [4, 7]. We used 3D-printed bone scaffolds to be implanted into the rib-periosteum-sealed cavity to successfully regenerate the required bone tissue. Further, we demonstrated the in vivo microenvironment plays a central role in bone tissue regeneration in a swine model using rib-sealed periosteal spaces [8]. With the availability of reliable animal models, many scholars have begun to study its molecular mechanism. Molecules involved in the periosteum during the guided bone regeneration in the periosteal closed chamber of the rib were detected, and differentially expressed genes and related pathways were found [9]. Microarray gene technique was used to detect gene expression and associated pathway function in guided bone regeneration for bone defects [9, 10]. The results showed that early immune and inflammatory responses and the Wnt pathway play important roles in bone regeneration [11, 12]. These studies opened the door to the molecular mechanism of guided bone regeneration, and laid a theoretical foundation for further intervention in this process [13].

NFAT (nuclear factor of activated T cells) is an important family of transcription factors which were first described in T cells [14]. The members of NFAT family include NFAT1-4 (also known as NFATc1–c4) and NFAT5. NFAT family plays an important role not only in the immune-inflammatory system, but also in development, regulation, and differentiation in other tissues [15, 16]. Activation of NFAT family of molecules can regulate a range of cellular activities. In previous studies, it was found that NFAT family regulates the activities of osteoclasts and osteoblasts in bone growth and development, thereby affecting bone formation [16, 17]. Therefore, we speculate that NFAT family may also play an important role in guiding bone regeneration, which has not been reported in the previous literature.

In this study, we created a long segment of bone defect-enclosed periosteal sheath in the swine rib as a microenvironment for guided bone regeneration. Microarray gene technique was used to detect the expression of NFAT family in the periosteum of regenerated bone and contralateral non-surgery control at different time points after surgery. The expression changes of NFAT family were dynamically recorded in the process of guided bone regeneration, and correlations among family genes were also analyzed.

## Materials and methods

This study was approved by the Animal Research Ethics Committee of Shanghai Ninth People’s Hospital Affiliated to Shanghai Jiaotong University School of Medicine (SH9H-2019-A645-1). The article was written in accordance with the ARRIVE (Animal Research: Reporting In Vivo Experiments) guidelines.

### Subjects and surgical procedures

Thirty-six healthy skeletally mature female swine with age ranging from 4 to 6 months were used in this study. The left ribs were used as the trauma group, and the contralateral ribs were used as the control group. We first touched the junction of the costal cartilage and bone by hand, where there is a raised tubercle that is easy to locate. A 3.0 cm incision was made from the tubercle of the fourth rib to the spine, and the skin and subcutaneous tissue were incised to expose the periosteum of the third, fourth, and fifth ribs. The periosteum was incised in the middle of the rib, and the subperiosteal rib was carefully stripped, and a 3 cm long rib was removed. The periosteum was re-sutured with 6-0 PDS sutures to form a closed periosteal sheath as a microenvironment for guided bone regeneration. We performed the same operation on the 3rd, 4th, and 5th ribs. Finally, the wound was sutured layer by layer.

### Specimen harvesting

At 1 day, 3 days, 1 week, 2 weeks, 1 month, 5 weeks, 3 months, 6 months, and 7 months after the surgery, the swine were sacrificed in batches. The periosteum of the guided regenerated bone in the trauma group and the periosteum of the same site in the control group were collected. Periosteum was used for subsequent next-generation sequencing, and some samples were used for hematoxylin–eosin staining to detect osteogenesis.

### Microarray analysis

The sequencing platform for this study was Illumina. Following the standard procedure for sequencing, the samples were subjected to total RNA extraction as well as total RNA quality testing. The Agilent 2100 Bioanalyzer was used to test the RNA integrity. The RNA concentration and purity tests were passed before the next step of the mRNA purification process. The total RNA was purified by the unique polyA structure in the total mRNA. The ion interruption was used to break the RNA to a fragment of about 300 bp in length. A fragment of 300 bp in length was selected. The first strand of cDNA was synthesized using RNA as template with 6-base random primers and reverse transcriptase, and the second strand of cDNA was synthesized using the first strand of cDNA as template. After library construction, PCR amplification was used for library fragment enrichment, followed by library selection based on fragment size, which was set at 450 bp. Subsequently, the libraries were quality checked by Agilent 2100 Bioanalyzer, and then the total library concentration and the effective library concentration were tested. Libraries containing different Index sequences were then mixed in proportion to the effective concentration of the library and the amount of data required for the library. The mixed libraries were uniformly diluted to 2 nM and denatured by alkali to form single-stranded libraries. After RNA extraction, purification and library construction, these libraries were sequenced using Next-Generation Sequencing technology based on the Illumina sequencing platform for Paired-end sequencing.

The raw downstream data were filtered, and the filtered high-quality sequences were compared to the reference genome of the species. Based on the comparison results, the expression of each gene was calculated. We used HTSeq statistics to compare to read count values on each gene as the original expression of the gene. Read count values are positively correlated with the true expression level of the gene, as well as the length and sequencing depth of the gene. In order to make gene expression levels comparable across genes and samples, expression was normalized using FPKM (Fragments Per Kilo bases per Million fragments), which is the number of fragments per kilobase length from a gene per million fragments. The read count of each gene is homogenized to obtain the baseMean value, which represents the expression level of the gene.

In this study, the baseMean values of NFAT family were extracted for further analysis. Nuclear factor of activated T cells 2 interacting protein (NFATC2IP) is 45 kDa NFAT-interacting protein, and regulates the magnitude of NFAT-driven transcription of a specific subset of cytokine genes. Although NFATC2IP is not a member of NFAT family, we also include its data in the analysis of NFAT family expression. The ratio of the baseMean value of the genes in the trauma group to that of the control group is the foldchange value. When the foldchange value is greater than 1, it indicates that the gene is up-regulated in the trauma group, and vice versa for down-regulation. The correlations of NFAT family expression within groups and between groups at various time points were analyzed. The correlation between the expression levels of NFAT family members at each different time point between groups was also analyzed.

### Statistical analysis

The SPSS statistical software (SPSS 14.0, Chicago, IL, USA) was used to perform statistical analysis. Data are described as mean ± standard error of mean (SE). Spearman correlation analysis was used to perform the correlation between different genes, different groups and different time points. *P* less than 0.05 was considered significant. The dynamic changes of baseMean and foldchange values were described with real data and smoothed graphs.

## Results

**Fig. 1:**
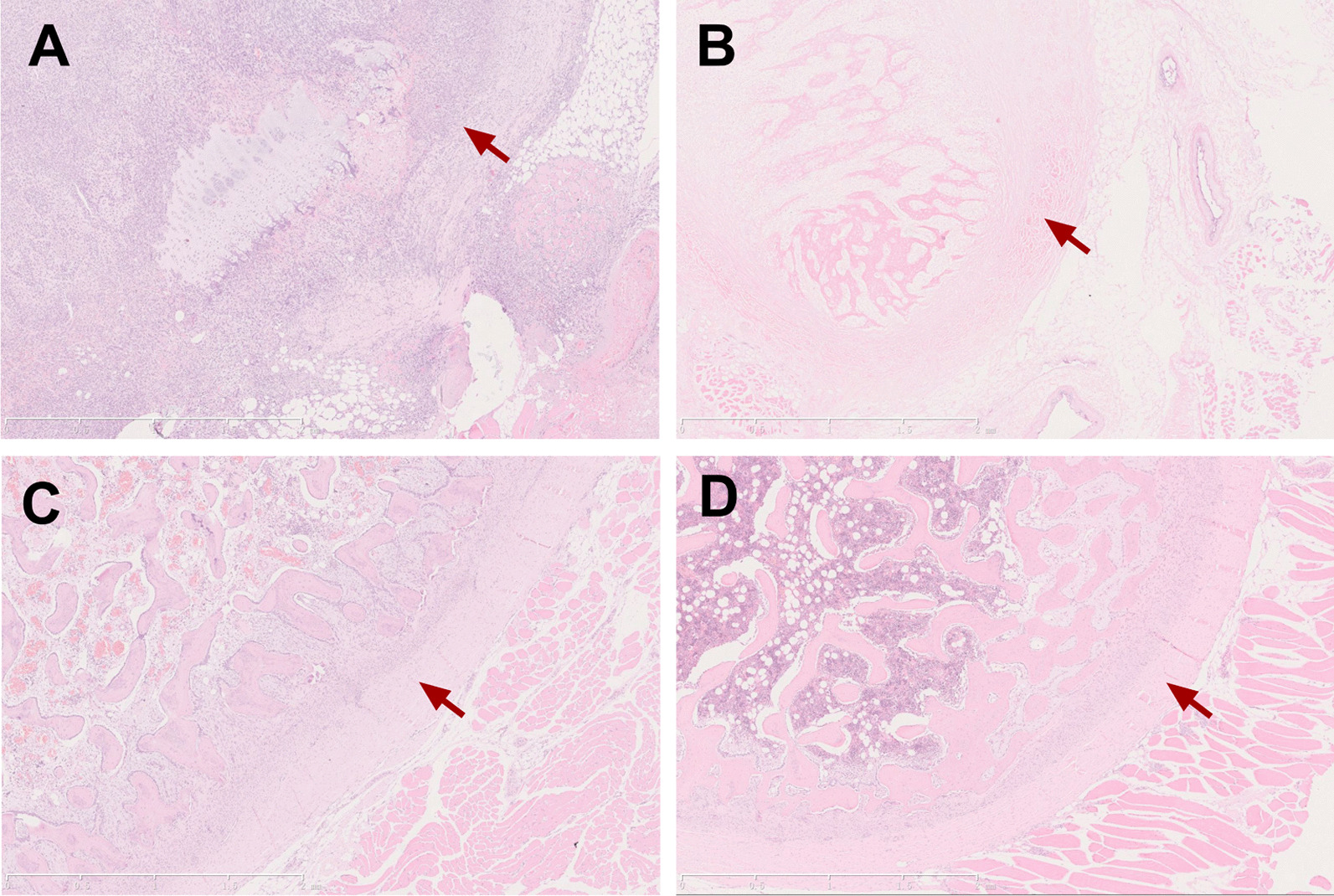
Hematoxylin–eosin staining of periosteum and regenerated bone. **a** The regeneration chamber was mainly composed of hematoma and inflammatory cells at postoperative third day. **b** Regenerated bone tissue was already visible at 1 week after surgery. **c** Within postoperative one-month period, complete bone regeneration had been achieved. **d** At 6 months after the surgery, the regenerated bone was well remodeled

All wounds showed no signs of infection. Hematoxylin–eosin staining showed that regenerated bone tissue was already visible at 1 week after surgery. Within postoperative one-month period, complete bone regeneration had been achieved. At 6 months after the surgery, the regenerated bone was well remodeled (Fig. 1).

**Fig. 2:**
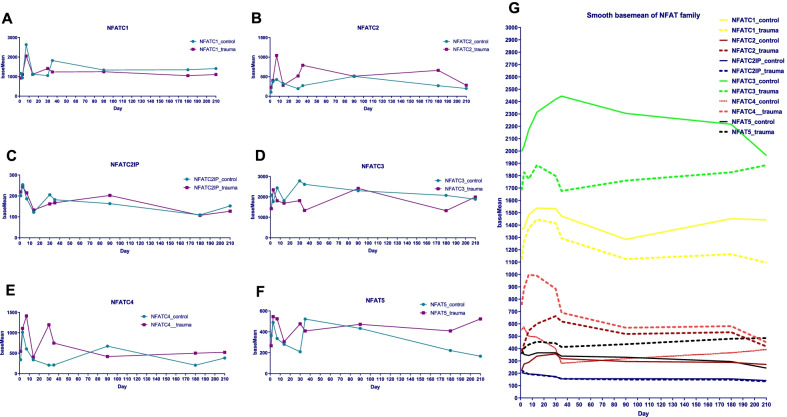
Dynamic changes of expression levels of NFAT family members at different time points. **a**–**f** Dynamic changes of expression levels of 6 NFAT family members. Within 1–1.5 months after the operation, the expression levels of each NFAT member in both the control group and the trauma group fluctuated greatly. **g** Smoothed basemean of NFAT family shows the expression level of NFATc3 is the highest, while the expression level of NFATC2IP is the lowest. The expression change trends of NFATc1 and NFATC2IP in trauma group were consistent with those of control group

The expression levels of each gene changed dynamically at each time point in both groups. The baseMean of NFATc1 in control and trauma group were 1425.0 ± 173.9 and 1251.0 ± 110.8, respectively, and both reached their highest values at 1 postoperative week (Fig. 2a). The baseMean of NFATc2 groups were 295.3 ± 42.0 and 522.7 ± 90.1 in both groups and reached their highest values at postoperative1 week and 3 months, respectively (Fig. 2b). The baseMean of NFATC2IP in the two groups were 175.0 ± 14.8 and 175.1 ± 15.8, respectively, and both peaked one week after surgery (Fig. 2c). The baseMean of NFATc3 were 2198.0 ± 120.3 and 1796.0 ± 134.9, respectively, and both reached their maximum values at 1 and 3 months postoperatively (Fig. 2d). The baseMean of NFATc4 in both groups were 441.4 ± 90.7 and 761.2 ± 126.4, respectively, and both peaked at 3 days and 1 week after surgery (Fig. 2e). The baseMean of NFAT5 in the two groups were 335.9 ± 42.4 and 436.6 ± 32.8, respectively, and both reached their maximum at 5 weeks and 3 days after surgery (Fig. 2f). By smoothing the baseMean value curve, the expression change trend of each gene in different periods can be displayed more clearly (Fig. 2g). The expression level of NFATc3 is the highest, while the expression level of NFATC2IP is the lowest. The expression change trends of NFATc1 and NFATC2IP in trauma group were consistent with those of control group.

**Fig. 3:**
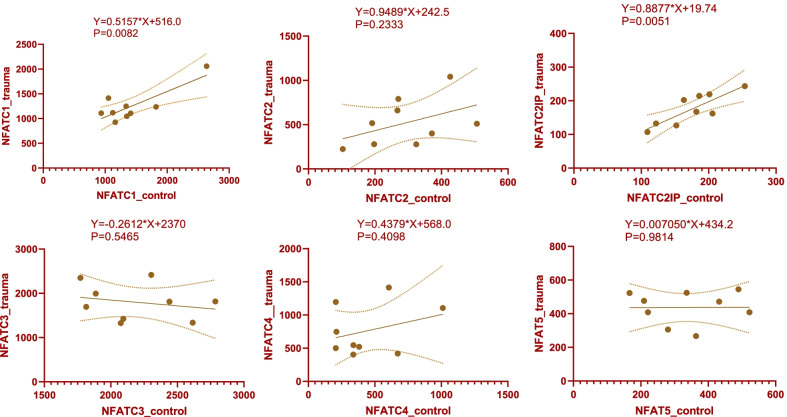
Linear correlation analysis on the expression levels of the same genes at different time points in the trauma group and the control group. NFATc3 was negatively correlated in the two groups, while the other five genes were positively correlated in the two groups. The dynamic expression levels of NFATc1 and NFATC2IP genes showed a significant positive correlation in the two groups

Linear correlation analysis was performed on the expression levels of the same genes at different time points between the two groups (Fig. 3). The results of correlation analysis showed that only NFATc3 was negatively correlated in the two groups, while the other five genes were positively correlated in the two groups. The dynamic expression levels of NFATc1 and NFATC2IP genes showed a significant positive correlation in the two groups (*Y* = 0.5157*X + 516.0, *P* = 0.0082; *Y* = 0.8877*X + 19.74, *P* = 0.0051, respectively).

**Fig. 4:**
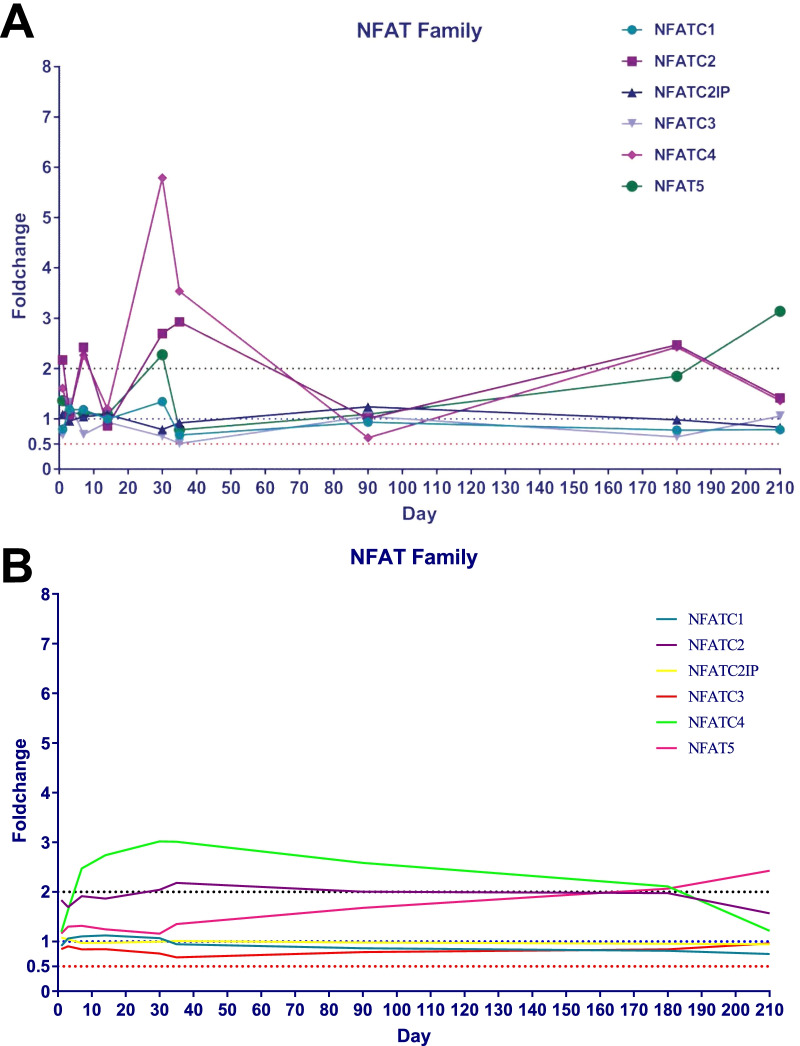
Dynamic changes of foldchange of each NFAT family member. **a** The foldchange changes dynamically with the most obvious fluctuations within 1–1.5 months. There are two obvious peaks in the foldchange of NFATc2 and NFATc4 genes, which are located at about one month and 6 months after surgery, respectively. **b** Smoothed line graph shows the foldchanges of NFATc2, NFATc4, and NFAT5 show a clear trend of over 1.0, while the dynamic trend of the other three genes was around 1.0

The ratio of the expression levels of the same gene in the trauma group relative to the control group was defined as the foldchange. The analysis results show that the foldchange changes dynamically at each time point (Fig. 4a). The line chart of foldchange shows that there are two obvious peaks in the foldchange of NFATc2 and NFATc4 genes, which are located at about one month and 6 months after surgery, respectively. After smoothing the foldchange line graph, the dynamic change trend of foldchange of each gene can be displayed more clearly. The foldchanges of NFATc2, NFATc4, and NFAT5 show a clear trend of over 1.0, while the dynamic trend of the other three genes was around 1.0 (Fig. 4b).

**Fig. 5:**
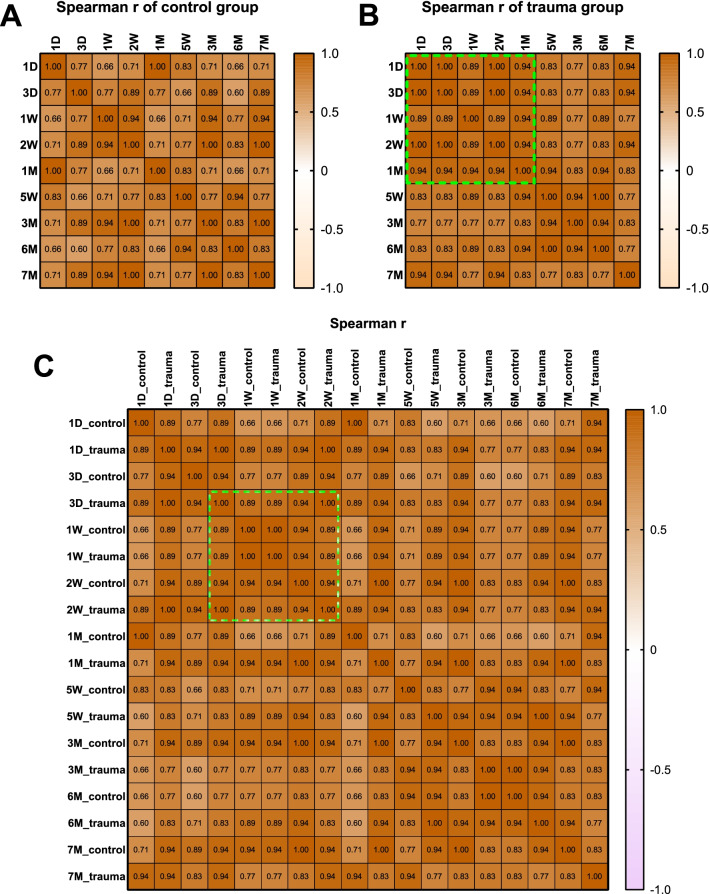
The correlation between the expression levels of six NFAT family members at different time points. **a** Spearman r value of control group. **b** In the trauma group, there were more significant correlations at each time point in the trauma group than in the control group and there was a centrally significant correlation between various time points between 1 day and 1 month, which is similar to the “pattern-block” phenomenon. **c** There was also a centrally significant correlation between 3 days and 2 weeks after surgery when two groups were analyzed together

The correlation between the expression levels of six NFAT family members at different time points was analyzed by Spearman correlation. In the control group, the expression levels of the NFAT family were positively correlated between various time points, and the time points of 2 weeks, 3 months, and 7 months are significantly correlated with most other time points (Fig. 5a). The *P* values of Spearman correlation analysis at different time points in the control group was shown in Additional file 1: Table S1. In the trauma group, the expression levels of the NFAT family at different time points also showed a positive correlation. There were more significant correlations at each time point in the trauma group than in the control group (Fig. 5b; Additional file 1: Table S2). And it can be found that there was a centrally significant correlation between various time points between 1 day and 1 month, which is similar to the “pattern-block” phenomenon [18, 19]. When the NFAT family expression at each time point in the trauma group and the control group were correlated together, it was found that there was also a centrally significant correlation between 3 days and 2 weeks after surgery (Fig. 5c; Additional file 1: Table S3).

**Fig. 6:**
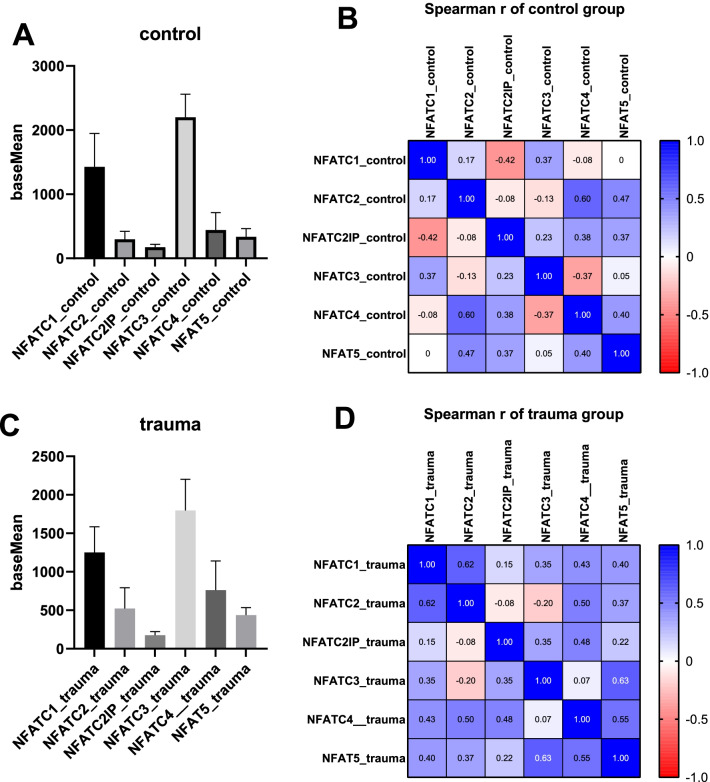
Expression correlation analysis among various NFAT family members. **a** The expression levels of each NFAT family member in the control group. **b** The expression correlation of each NFAT family in the control group. **c** The expression levels of each NFAT family member in the trauma group. **d** The expression correlation of each NFAT family in the trauma group

The expression levels of each NFAT family member in the trauma group and the control group were analyzed, respectively. In the control group, the expression level of NFATc3 was the highest, while the expression level of NFATC2IP was the lowest (Fig. 6a). The expression levels of each NFAT family in the control group included positive correlation and negative correlation, but no significant correlation (Fig. 6b). While in the trauma group, the expression level of NFATc3 was the highest, and the expression level of NFATC2IP was the lowest, which were consistent with those of control group (Fig. 6c). Similarly, the correlations between the expression levels of various NFAT family members in the trauma group also included positive and negative correlations, but none of them reached significant correlations (Fig. 6d).

## Discussion

This study examines the dynamic changes in the expression of the NFAT family in the periosteum during guided bone regeneration in vivo. More specifically, the expression of NFAT family in the periosteum of the trauma group and the control group was compared in the early, middle and late postoperative period. Correlation analysis was carried out for the expression relationship between each NFAT family members between different time points and different groups.

In previous studies, the researchers used a rat skull defect model and covered the skull defect with biofilms of different materials to form a relatively closed regenerative microenvironment that guides bone regeneration and prevents soft tissue ingrowth [3, 10, 20]. Subsequently, molecular expression assays were performed on the regenerated bone tissue to investigate the possible molecular mechanisms in this guided bone regeneration process. The periosteal closed regeneration chamber model established in this study is in line with the principle of bone regeneration. In previous studies, the reliability of the model has also been verified [8, 9]. In this study, we focused on the molecular expression changes of the periosteum during bone regeneration, so as to explore the molecular mechanism involved in the periosteum, which is different from previous studies on the changes of bone regeneration molecules [10, 20]. In addition, the model can guide bone regeneration for bone and joint defects, and construct the corresponding regenerated bone shape, which can not only be repaired in situ, but also can be used to repair bone and joints in other parts [9]. We have also successfully used this model to regenerate mandibles and joints for repairing and reconstructing defects in our previous preconstruction studies [4, 7, 8].

In the process of guiding bone regeneration, the early immune and inflammatory responses play an important role [5, 13, 21]. The metabolic activities of osteoblasts and osteoclasts play a key role in the early, middle, and late stages. The NFAT family is involved in immune and inflammatory responses, and also plays an important role in regulating the metabolism of osteoblasts and osteoclasts [22, 23]. Therefore, understanding the specific expression changes of the NFAT family in the process of guided bone regeneration is of great significance for the study of the molecular mechanism involved.

At different time points, the expression of each NFAT family member in the trauma group and the control group fluctuated greatly. This indicates that as a control group, the molecular metabolism of the periosteum is not static and is also affected by surgery in other parts. This result also raises a new question of whether, when we choose the control group, we should choose the periosteum of the unoperated site of the same animal or the periosteum of the baseline period, which needs to be further explored. Among the expression levels of each NFAT family member, the level of NFATc3 is the highest, and the expression level of NFATC2IP is the lowest. Within 1–1.5 months after surgery, the expression levels of each member fluctuated the most, indicating that the NFTA family was involved in various metabolic reactions in the body during this period. The NFAT family is a product expressed in activated T cells in the immune system, and in the early post-operative period [24]. T cells are involved in immune and inflammatory responses in the body [14, 15]. During this process, the osteogenic activity is also very active, which can also explain the large change of NFAT family at this stage.

In previous, a mouse model of muscular dystrophy was established to investigate the function of NFAT in muscle injury, regeneration and repair, and it revealed that the myoferlin, a member of the ferlin family, was regulated bye NFAT in the muscle regeneration [25]. Ge X. et al. found that NFATc1 restricts the proliferation and chondrogenesis of osteochondroma precursors and NFATc2 preferentially inhibits chondrocyte hypertrophy and osteogenesis, which suggests that skeletal diseases characterized by defective or exaggerated osteochondral growth may be treated by regulating NFAT activity [26]. Farrera-Hernández A et al. demonstrated that non canonical WNT5A-Ca2 + -CaN-NFAT signalling plays a key role during embryonic digit development in vivo promoting the competence for chondrogenic signals and also acts as a permissive factor for chondrogenesis independently of cell death mechanisms.

The expression levels of the same NFAT family members were positively correlated in both trauma and control groups, except for NFATc3. The expression levels of NFATc1 and NFATC2IP in the trauma group were significantly positively correlated with the expression levels of the corresponding genes in the control group. We speculate these two genes may affect the expression levels of the same genes on the contralateral side through the action of the blood circulation system, and the molecules mediated in the blood system need to be further studied. When using foldchange to evaluate the relative expression of each NFAT family member, it was found that the foldchange curve were very different from the baseMean curve. Similarly, the foldchange values of most NFAT family members within 1–1.5 months have relatively large fluctuations, especially the values of NFATc2 and NFATc4 have more than 2 times for a long period of time, and are accompanied by two very obvious peaks. This also suggests that these two genes may play specific roles in the guided bone regeneration from trauma, relative to controls. The foldchange values of NFATC2IP and NFATc3 were around 1.0, which may indicate that after surgical trauma, these two genes play a general role in the periosteum of the whole body, but have no obvious specific effect on the trauma.

When studying the correlation of NFAT family expression at different time points, it was found that in both groups, the expression levels at different time points were positively correlated. However, the significant correlation at each time point in the trauma group was significantly stronger than that in the control group. In the trauma group, the expression of NFAT family at each time point within 1 month showed a significant positive correlation, but this phenomenon was not observed in the control group. When both groups were analyzed simultaneously, this clustered and significant correlation was found to occur over a period of 3 days to 2 weeks, which is similar to the “pattern-block” phenomenon. This phenomenon was first reported in the study of the clinical manifestations and nucleotide polymorphisms of fingers [18, 19]. Scholars found that the clinical phenotypes and nucleotide polymorphisms between the middle fingers had a more significant correlation [18]. The appearance of this phenomenon in this study suggests that in the time period within 1 month, the expression of NFAT families is more significantly correlated. Therefore, when we study the role of the NFAT family in the process of guiding bone regeneration, we should focus on this time period. Within the same group, there was no significant correlation between NFAT family members, including the control group and the trauma group. It is speculated that although some of these gene families have similar effects, the interaction between them may not be obvious. In particular, it is of concern that the expression levels between NFATc2 and NFATC2IP also did not show a significant correlation, indicating that there are more complex factors involved in the regulation between the two molecular.

In the future, research on the specific mechanism of the NFAT family involved in guiding bone regeneration may need to rely on in vitro experiments and in vivo experiments in mouse models. In in vitro studies, we could verify at the cellular level the possible molecular roles of individual NFAT family members in guiding bone regeneration. The final functional verification and intervention effect still need to go back to in vivo testing. At present, studies on conditional mice with NFATc1 and NFATc2 gene knockout have been reported [15, 16, 27]. After gene knockout, it was found that the osteogenic and cartilage-forming abilities of the mice were significantly lower than those of normal mice [22, 23]. This indicates that NFATc1 and NFATc2 are very important regulators in osteogenesis and chondrogenesis. The functions of NFAT family members in this study, especially NFATc2 and NFATc4, which are significantly higher expressed than those in the control group, can also be studied by in vitro experiments. Similarly, we can use Cre-specific gene knockout of this gene, and further verify and intervene in mice in vivo experiments to explore the mechanism of its involvement in guiding bone regeneration. Moreover, the establishment of a periosteal regeneration chamber-guided bone regeneration model in mice has been reported successfully [28], which is a very good foundation for our further research.

In this study, we established a periosteal regeneration chamber model in swine to simulate guided bone regeneration in humans. The expression changes of NFAT family members were dynamically described by gene detection of periosteum of regenerated bone and non-operated control periosteum at 9 time points between 1 day and 7 months. Previous studies of the NFAT family have reported that NFAT plays an important role in inflammation, immune response, and osteogenesis and chondrogenesis, and these metabolic processes also play an important role in guiding bone regeneration. The actual dynamic expression levels of each NFAT family member in the two groups fluctuated to a certain extent, and the foldchange curve provided the gene expression of the trauma group at the same time point relative to the control group, and the two different analysis results had many differences. Therefore, when we study the expression of molecules in animal models, we should not only use the non-intervention part as a control, but also use the baseline point as a control. Such analysis may provide more comprehensive information about the relative expression of the molecules studied. Within 1–1.5 months after surgery, the expression of NFAT family members fluctuates greatly, and at this stage, the correlation of gene expression at each time point is more significant. Therefore, we should focus on the change of this time stage in studying the mechanism of NFAT family in guided bone regeneration. The relative expression levels of NFATc2 and NFATc4 in the trauma group were significantly higher than those in the control group, indicating that these two genes may play an important role in guiding bone regeneration. In future studies, we can verify the functions by in vitro experiments. In vivo study, we can use knockout mice to design interventions to study the specific mechanisms by which these genes are involved in guiding bone regeneration.

## Supplementary Information

**Supplementary file 1. Table S1**: P values of Spearman correlation analysis of NFAT family at different time points in the control group. **Table S2**: P values of Spearman correlation analysis of NFAT family at different time points in the trauma group. **Table S3**: P values of Spearman correlation analysis of NFAT family at different time points in both groups.

## Abbreviation

NFAT: Nuclear factor of activated T cells
