# Supplementary material for: Dynamic transcriptome analysis of NFAT family in guided bone regeneration with occlusive periosteum in swine model
Source: J Orthop Surg Res. 2022 Jul 26;17:364. doi: 10.1186/s13018-022-03252-9 (PMC9327338; doi:10.1186/s13018-022-03252-9)
Supplement: Supplementary file 1 — Table S1: P values of Spearman correlation analysis of NFAT family at different time points in the control group. Table S2: P values of Spearman correlation analysis of NFAT family at different time points in the trauma group. Table S3: P values of Spearman correlation analysis of NFAT family at different time points in both groups. [file 13018_2022_3252_MOESM1_ESM.pdf]

Supplementary table 1. P values of Spearman correlation analysis of NFAT family at different time points in the control group.

**Time points**	1D	3D	1W	2W	1M	5W	3M	6M	7M
1D		0.102778	0.175	0.136111	0.002778	0.058333	0.136111	0.175	0.136111
3D	0.102778		0.102778	0.033333	0.102778	0.175	0.033333	0.241667	0.033333
1W	0.175	0.102778		0.016667	0.175	0.136111	0.016667	0.102778	0.016667
2W	0.136111	0.033333	0.016667		0.136111	0.102778	0.002778	0.058333	0.002778
1M	0.002778	0.102778	0.175	0.136111		0.058333	0.136111	0.175	0.136111
5W	0.058333	0.175	0.136111	0.102778	0.058333		0.102778	0.016667	0.102778
3M	0.136111	0.033333	0.016667	0.002778	0.136111	0.102778		0.058333	0.002778
6M	0.175	0.241667	0.102778	0.058333	0.175	0.016667	0.058333		0.058333
7M	0.136111	0.033333	0.016667	0.002778	0.136111	0.102778	0.002778	0.058333	

P value less than 0.05 was highlighted.

Supplementary table 2. P values of Spearman correlation analysis of NFAT family at different time points in the trauma group.

**Time points**	1D	3D	1W	2W	1M	5W	3M	6M	7M
1D		0.002778	0.033333	0.002778	0.016667	0.058333	0.102778	0.058333	0.016667
3D	0.002778		0.033333	0.002778	0.016667	0.058333	0.102778	0.058333	0.016667
1W	0.033333	0.033333		0.033333	0.016667	0.033333	0.102778	0.033333	0.102778
2W	0.002778	0.002778	0.033333		0.016667	0.058333	0.102778	0.058333	0.016667
1M	0.016667	0.016667	0.016667	0.016667		0.016667	0.058333	0.016667	0.058333
5W	0.058333	0.058333	0.033333	0.058333	0.016667		0.016667	0.002778	0.102778
3M	0.102778	0.102778	0.102778	0.102778	0.058333	0.016667		0.016667	0.058333
6M	0.058333	0.058333	0.033333	0.058333	0.016667	0.002778	0.016667		0.102778
7M	0.016667	0.016667	0.102778	0.016667	0.058333	0.102778	0.058333	0.102778	

P value less than 0.05 was highlighted.

Supplementary table 3. P values of Spearman correlation analysis of NFAT family at different time points in both groups.

Time points	1D_control	1D_trauma	3D_control	3D_trauma	1W_control	1W_trauma	2W_control	2W_trauma	1M_control	1M_trauma	5W_control	5W_trauma	3M_control	3M_trauma	6M_control	6M_trauma	7M_control	7M_trauma
1D_control		0.033333	0.102778	0.033333	0.175	0.175	0.136111	0.033333	0.002778	0.136111	0.058333	0.241667	0.136111	0.175	0.175	0.241667	0.136111	0.016667
1D_trauma	0.033333		0.016667	0.002778	0.033333	0.033333	0.016667	0.002778	0.033333	0.016667	0.058333	0.058333	0.016667	0.102778	0.102778	0.058333	0.016667	0.016667
3D_control	0.102778	0.016667		0.016667	0.102778	0.102778	0.033333	0.016667	0.102778	0.033333	0.175	0.136111	0.033333	0.241667	0.241667	0.136111	0.033333	0.058333
3D_trauma	0.033333	0.002778	0.016667		0.033333	0.033333	0.016667	0.002778	0.033333	0.016667	0.058333	0.058333	0.016667	0.102778	0.102778	0.058333	0.016667	0.016667
1W_control	0.175	0.033333	0.102778	0.033333		0.002778	0.016667	0.033333	0.175	0.016667	0.136111	0.033333	0.016667	0.102778	0.102778	0.033333	0.016667	0.102778
1W_trauma	0.175	0.033333	0.102778	0.033333	0.002778		0.016667	0.033333	0.175	0.016667	0.136111	0.033333	0.016667	0.102778	0.102778	0.033333	0.016667	0.102778
2W_control	0.136111	0.016667	0.033333	0.016667	0.016667	0.016667		0.016667	0.136111	0.002778	0.102778	0.016667	0.002778	0.058333	0.058333	0.016667	0.002778	0.058333
2W_trauma	0.033333	0.002778	0.016667	0.002778	0.033333	0.033333	0.016667		0.033333	0.016667	0.058333	0.058333	0.016667	0.102778	0.102778	0.058333	0.016667	0.016667
1M_control	0.002778	0.033333	0.102778	0.033333	0.175	0.175	0.136111	0.033333		0.136111	0.058333	0.241667	0.136111	0.175	0.175	0.241667	0.136111	0.016667
1M_trauma	0.136111	0.016667	0.033333	0.016667	0.016667	0.016667	0.002778	0.016667	0.136111		0.102778	0.016667	0.002778	0.058333	0.058333	0.016667	0.002778	0.058333
5W_control	0.058333	0.058333	0.175	0.058333	0.136111	0.136111	0.102778	0.058333	0.058333	0.102778		0.058333	0.102778	0.016667	0.016667	0.058333	0.102778	0.016667
5W_trauma	0.241667	0.058333	0.136111	0.058333	0.033333	0.033333	0.016667	0.058333	0.241667	0.016667	0.058333		0.016667	0.016667	0.016667	0.002778	0.016667	0.102778
3M_control	0.136111	0.016667	0.033333	0.016667	0.016667	0.016667	0.002778	0.016667	0.136111	0.002778	0.102778	0.016667		0.058333	0.058333	0.016667	0.002778	0.058333
3M_trauma	0.175	0.102778	0.241667	0.102778	0.102778	0.102778	0.058333	0.102778	0.175	0.058333	0.016667	0.016667	0.058333		0.002778	0.016667	0.058333	0.058333
6M_control	0.175	0.102778	0.241667	0.102778	0.102778	0.102778	0.058333	0.102778	0.175	0.058333	0.016667	0.016667	0.058333	0.002778		0.016667	0.058333	0.058333
6M_trauma	0.241667	0.058333	0.136111	0.058333	0.033333	0.033333	0.016667	0.058333	0.241667	0.016667	0.058333	0.002778	0.016667	0.016667	0.016667		0.016667	0.102778
7M_control	0.136111	0.016667	0.033333	0.016667	0.016667	0.016667	0.002778	0.016667	0.136111	0.002778	0.102778	0.016667	0.002778	0.058333	0.058333	0.016667		0.058333
7M_trauma	0.016667	0.016667	0.058333	0.016667	0.102778	0.102778	0.058333	0.016667	0.016667	0.058333	0.016667	0.102778	0.058333	0.058333	0.058333	0.102778	0.058333	

P value less than 0.05 was highlighted.
